# Individual year-round movement patterns of the GPS-tracked Grey Herons (*Ardea cinerea*) in Central Europe: a multi-year case study

**DOI:** 10.7717/peerj.21659

**Published:** 2026-09-04

**Authors:** Brygida Manikowska-Ślepowrońska, Karolina Cieślińska, Krzysztof Ślepowroński, Ewa Poślińska, Joachim Siekiera, Michał Goc, Lech M. Iliszko, Dariusz Jakubas

**Affiliations:** 1 Department of Vertebrate Ecology and Zoology, Faculty of Biology, University of Gdansk, Gdańsk, Poland; 2 Regional Directorate for Environmental Protection in Opole, Opole, Poland; 3 Rzeczna 17, .Zywocice, Poland

**Keywords:** Migration, GPS-GSM telemetry, Dispersal, Stop-overs, Site-fidelity, Movement ecology

## Abstract

**Background:**

The year-round migratory behaviour of Grey Herons (*Ardea cinerea*) breeding in Central Europe is recognized; however, individual consistency in their movement patterns remains poorly known. In this study, we present movements of three adult Grey Herons breeding in Northern Poland and Eastern German global positioning system (GPS)-tracked between 2012 and 2020.

**Methods:**

We analyzed annual movement trajectories, focusing on migration distances, fidelity to breeding and wintering grounds, and the use of stop-over sites. We performed an Analysis of Variance from Summary Data (ANOVA) on summary data to compare migratory parameters across different Eurasian populations and age groups.

**Results:**

We identified four main phases in the annual cycle of all studied individuals: breeding, wintering, spring and autumn migration. Our data reveal high inter-individual variation in migratory strategies. Although two individuals changed their wintering sites from year to year, all studied ones exhibited strict fidelity to the same breeding grounds across consecutive seasons. Migration distances ranged from 175 to 1,758 km; notably, the individual breeding in Eastern Germany covered the shortest distance (mean ± SD: 221 ± 51.8 km). We observed substantial inter-individual variation in winter site fidelity, migration distances, and stop-over numbers within this Central European Grey Heron population. The herons selected the shortest migration paths during spring, but not during autumn. While the broad migratory characteristics—specifically the number of stop-overs (3.0 ± 2.8), migration distance (1,220 ± 580.4 km), and migration duration (9 ± 9 days)—closely match findings from other populations, the tracked herons in our study made fewer stop-overs during autumn migration compared to the values reported in the literature.

**Conclusions:**

We observed inter-individual variations in the annual movement patterns of the Grey Heron, encompassing the timing of the four annual cycle phases, the range of distances covered annually and the birds’ fidelity to areas used as breeding or wintering grounds. Our report provides the first long-term, high-resolution insights into the year-round movements of GPS-tracked Grey Herons from Central Europe, emphasizing that specific individuals employ diverse and flexible migratory strategies, even within the same breeding region.

## Introduction

Research covering the full annual cycle of birds increasingly emphasizes how different types of movement influence avian survival and population dynamics. The literature classifies these avian movements according to various purposes, such as: (1) routine day-to-day movements centred on the place of residence; (2) dispersal movements from natal sites; (3) dispersive migrations, including post-breeding movements in any direction; and (4) true migrations, where individuals perform regular return movements at approximately the same time each year, often to specific destinations (Dingle & Drake, 2007; Newton, 2010). Ornithologists investigate these movement types using various methods, including direct observations, ring recoveries, archival tags, or remote tracking *via* radar, radio, or satellite (Newton, 2010). The last category includes tracking based on the Global Positioning System (GPS), which provides highly reliable, high-quality data on bird movements across various avian species (Higuchi & Pierre, 2005; Kang et al., 2016; Ye et al., 2018; Gu et al., 2019; Liu et al., 2020; Lim et al., 2021; Morganti et al., 2021; Huang et al., 2022). GPS-tracking significantly enhances the understanding of avian migrations, particularly by identifying the locations of wintering grounds and stop-over sites. Furthermore, this technology allows for the exploration of other critical ecological and life history aspects, such as establishing home range sizes, determining multi-scale habitat selection, evaluating site fidelity, and identifying the precise location of night roosts along with measuring the distances covered to and from them (Van der Winden et al., 2012; Ledwoń & Betleja, 2014; Ye et al., 2018; Lumpkin et al., 2023; Manikowska-Ślepowrońska et al., 2025).

Annual movements of the Grey Heron (*Ardea cinerea*), despite being the most common species of heron in Europe (Cramp, 1998), have been studied in relatively few studies (*e.g.*, Rydzewski, 1956; Manikowska-Ślepowrońska, Mokwa & Jakubas, 2018). Within Central Europe, ringing recovery data indicate clear latitudinal differences in migration patterns. Specifically, northern populations, such as those in Poland, typically perform long-distance migrations towards Mediterranean countries (*e.g.*, Spain, France, and Italy), whereas populations from lower or more western latitudes, exhibit shorter migration distances or a higher frequency of non-migratory and dispersive individuals (Manikowska-Ślepowrońska, Mokwa & Jakubas, 2018). As a widespread waterbird breeding across Europe, Africa, and Asia, this species migrates in small flocks, occasionally forming mixed flocks with other Ardeids (Suchantke, 1960), and usually travels at night (Cramp, 1998). Autumn migration—generally oriented in SW direction in Europe, W Asia and S & SW direction in S Asia—starts in early September and lasts until the end of October, extending into late November in N Europe, while only a small fraction travels to S Africa or remains strictly sedentary (Voisin, 1991; Cramp, 1998). The majority of European populations of the Grey Heron spend the winter in Europe. Spring migration (NE direction) starts in February, and the birds reoccupy their colonies usually in March (April for N Europe) (Voisin, 1991; Cramp, 1998; Hancock & Kushlan, 2010; Manikowska-Ślepowrońska, Mokwa & Jakubas, 2018; Manikowska-Ślepowrońska, Mokwa & Jakubas, 2021). Although many Grey Herons return to their natal heronry, some individuals select another colony located nearby or far from the natal one (Voisin, 1991; Manikowska-Ślepowrońska, Mokwa & Jakubas, 2021). Sometimes, adult individuals also wander very far, *e.g.*, to Greenland, Iceland, or North America (Hancock & Kushlan, 2010).

Although biology of the Grey Heron is well documented (Milstein, Prestt & Bell, 1970; Voisin, 1991; Cramp, 1998), there are only a few studies focus on its migration. Most of these have been based mainly on ringing recoveries and described patterns of dispersion, migration, and recognized stop-over sites and wintering quarters. These studies often take the form of specific analyses (Rydzewski, 1956; Manikowska-Ślepowrońska, Mokwa & Jakubas, 2018; Manikowska-Ślepowrońska, Mokwa & Jakubas, 2021) or ringing atlases (Finlayson, 1992; Fransson & Pettersson, 2001; Wernham et al., 2002; Bakken, Runde & Tjřrve, 2003; Bønløkke et al., 2006; Cepák et al., 2008; Finlayson, 2010). So far, studies using GPS-tracking have focused on home range during breeding, post-breeding and/or natal dispersion, and migration distances. They were performed solely on Asian populations breeding in the Russian Far East, South Korea, and China (Suchantke, 1960; Kim et al., 2015; Ye et al., 2018; Lim et al., 2021; Tiunov et al., 2022). The only existing European studies utilized radio-tracked individuals based on traditional telemetry, focusing strictly on foraging strategies during the breeding seasons in France (Marion, 1989) and Belgium (Van Vessem & Draulans, 1987). Consequently, fundamental aspects of year-round Grey Heron movements remain poorly understood (*e.g.*, timing of migration, presence/absence of stop-over sites).

Understanding these annual movements is critical not only for designing effective international conservation strategies for wetland-dependent species but also for predicting how migratory birds adapt to rapid climate change. Knowledge of the annual movement may be also important for wildlife management authorities as in some regions the Grey Heron is considered as a conflict species (Manikowska-Ślepowrońska, Szydzik & Jakubas, 2016). To fill this gap of knowledge, our study investigates, for the first time in Europe, the annual movements of GPS-tracked adult Grey Herons. Our main aims were to: (1) describe movements during particular phases of the annual life cycle (wintering, breeding, spring and autumn migrations), including migration routes and stop-over sites of the Central European population; (2) compare life phase durations and migration distances between individuals breeding in N Poland and E Germany; and (3) summarize the movement characteristics of European and Asian populations based on data reported in the literature.

We expected that:

(1) Given the results of previous ringing-based studies (Rydzewski, 1956; Manikowska-Ślepowrońska, Mokwa & Jakubas, 2018; Manikowska-Ślepowrońska, Mokwa & Jakubas, 2021) and harsh winter weather in Central European breeding areas (frozen shallow waterbodies and marshes that serve as the main foraging grounds; Jakubas, 2011), all studied individuals would migrate;

(2) Given that some individuals from Central Europe migrate far south for the winter (Manikowska-Ślepowrońska, Mokwa & Jakubas, 2018), and long-distance flights demand high energy, the birds might minimize journey costs by dividing the trip into several shorter flights (Alerstam, 2001), utilizing a few stopover sites *en route* to their wintering or breeding grounds;

(3) Given that ringing recovery data showed distinct migration distances between these two populations (Manikowska-Ślepowrońska, Mokwa & Jakubas, 2018), some characteristics, such as the duration of life phases or migration distances, would differ between individuals breeding in N Poland and E Germany;

(4) Given that Grey Herons experience different climatic conditions in their European and Asian breeding areas, the Asian and European populations likely developed distinct migratory movement characteristics.

## Materials & Methods

### Field study

#### Logger deployment

We investigated the movement data of three adult Grey Herons equipped with GPS-GSM loggers (which transmitted daily recorded data *via* the GSM network) captured in N and SW Poland. We caught the first individual (hereafter POL-KR) using a mist-net on 12 May 2012 in the breeding colony at Kąty Rybackie on the Vistula Spit, N Poland (54.350483, 19.196183), during the egg incubation stage. We deployed a DUCK-3 GPS-GSM logger with a solar panel (ECOTONE, Sopot, Poland; 56 × 27 × 20 mm, approx. 27 g) on this individual. We captured the second bird (hereafter POL-OL) on 21 July 2015 in a natural breeding colony located at Gdańsk-Oliwa ZOO, N Poland (54.412815, 18.532933), at the end of the chick-rearing period. We baited this individual with fish to lead it into a loop-trap made of fishing monofilament line (diameter 0.35 mm) placed on the ground, which we pulled and tightened around the bird’s leg for a successful capture. We deployed a SAKER H GPS-GSM logger with a solar panel (ECOTONE, Sopot, Poland; 58 × 27 × 28 mm, 20 g) on POL-OL. Given the behaviour of POL-KR and POL-OL after logger deployment—specifically their actions as central-place foragers repeatedly returning to the same spot in the colony—we assumed that both birds were active breeders.

**Figure 1:**
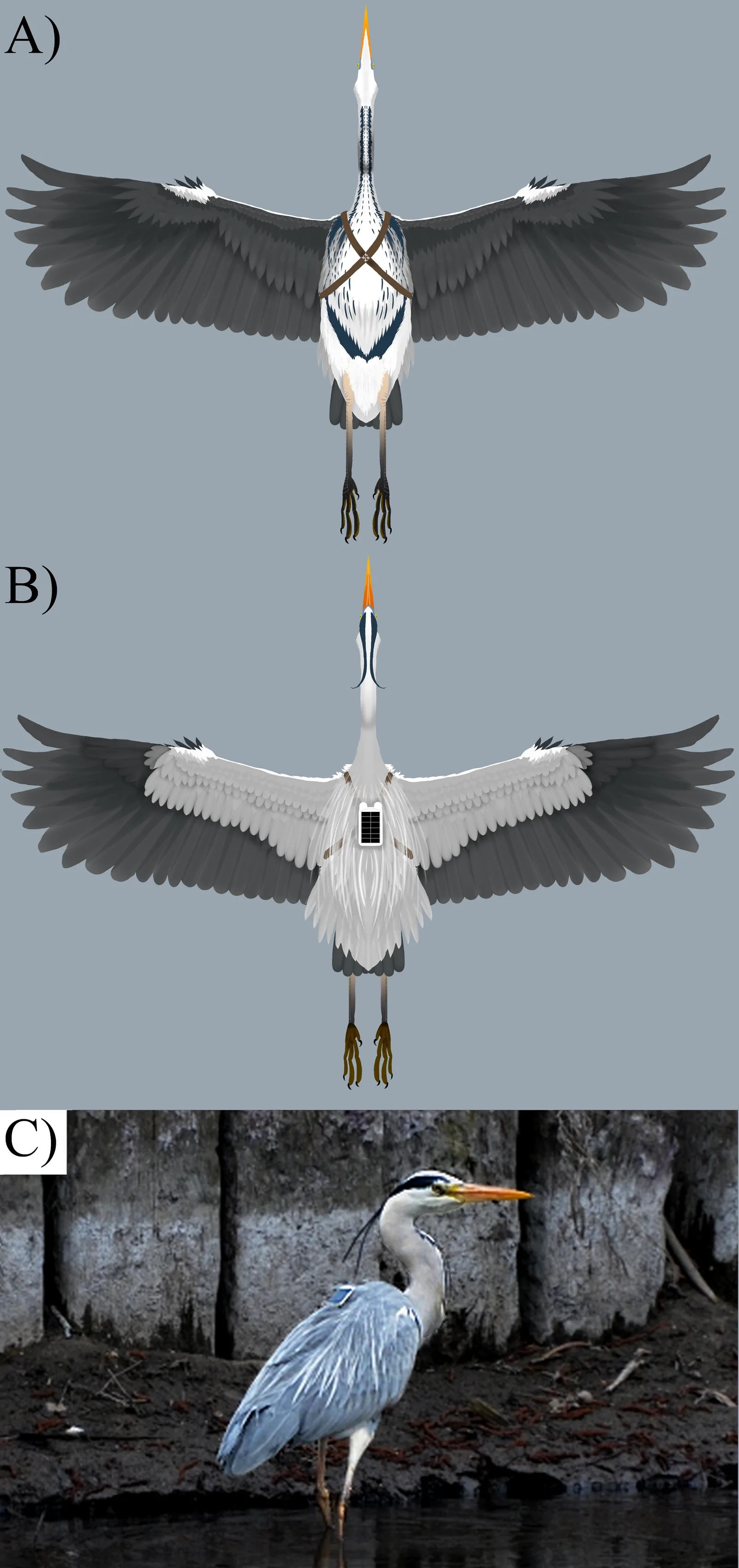
Images show location of the harness with GPS-logger on the studied Grey Herons. (A) Dorsal view. (B) Ventral view. (C) The POL-OL breeding in N Poland with GPS-logger mounted using the backpack harness photographed in the foraging area (small pond) during the breeding season. Art by K.C., photo by D.J.

We captured the third individual (hereafter GER-NR) by hand on 20 September 2018 at the Nysa Reservoir, SW Poland (50.450966, 17.217782), showing symptoms of poisoning. After curing at the asylum, we deployed a DUCK-4 GPS-GSM logger with a solar panel (ECOTONE, Sopot, Poland; 60 × 27 × 29 mm, 25 g) on 1 November 2018 and released the bird at the capture location. We fitted all of transmitters onto the birds’ backs using a lightweight Teflon backpack harness (10 g) (Fig. 1). We did not determine sex of the captured individuals. Since literature estimates the mean body mass of adults at 1,361 g for females and 1,505 g for males (Cramp, 1998), the total weight of the deployed device and its attachment constituted 1.3–1.5% and 1.5–2.2% of the body mass for females and males, respectively. These values remain well below the generally recommended 3% threshold (Kenward, 2001). We tracked Grey Herons until the signal was lost, presumably due to transmitter loss or device failure. We captured, handled, and tagged the birds with GPS loggers in full compliance with Polish law under an official ringing permission.

The Ministry of Science and Higher Education National Ethics Committee for Animal Experiments exempted this study from the requirement for specific ethical approval (Manikowska-Ślepowrońska et al., 2025), in accordance with Resolution No. 8/2006 of July 11, 2006, regarding the least painful methods of marking animals. Based on Art. 28, Section 2, Item 1 of the Act of January 21, 2005, point 8 specifically clarifies that marking with radio-telemetric transmitters attached externally to the animal’s body using collars, harnesses, tapes, or glue does not constitute an experimental procedure requiring specific IACUC approval, provided the transmitter’s weight does not exceed 5% of the animal’s body weight. The described field studies did not require any additional specific permits.

#### GPS-GSM-logger settings and work

We initially set the GPS logger sampling rate for POL-KR to 1 h. However, in reality, the logger recorded GPS fixes several times per day (ranging from 1 to 12 times per 24 h), depending on the battery voltage. The device did not record any data between 8 November 2012 and 5 March 2013 due to a low battery level, and we received the final location transmission on 28 October 2013. For POL-OL, we set the GPS sampling rate to 1 h for the period from 21 July 2015 to 4 October 2015; later, to save battery life, and ensure uninterrupted multi-year tracking during periods of limited solar charging, we remotely changed this interval to 3 h from 5 October 2015 to 5 June 2018. We configured the GPS sampling rate for GER-NR to 1 h and collected data exclusively between 6 am and 5 pm (to save battery life; see above). When the battery voltage dropped too low, the device recorded locations between 2 to 10 times per day. The device failed to record data in February 2020, and we obtained the last recorded location on 21 April 2020. It is highly likely that frequent periods of dense cloud cover and shorter daylight hours in winter caused the battery voltage to drop, resulting in these tracking gaps.

### Data analyses

All statistical analyses were conducted in R software ver. 4.1.3 (R Core Team, 2022).

#### Segmentation of movement data

To divide all recorded GPS locations into spatio-temporal periods reflecting the main phases of the avian annual life cycle, we used the segmentation implemented in the *segclust2* package (Patin et al., 2018). In this context, each calculated ‘segment’ biologically represents a distinct phenological phase of the avian annual cycle, defined by consistent spatial and behavioural patterns. We divided all tracking records based on GPS coordinates, employing the algorithm to find the best matching set of segment numbers for each individual by using a Dynamic Programming approach. We chose the number of segments using Lavielle’s criterion procedure (Lavielle, 2005), which locates a rupture in the penalized likelihood. We set the minimum length of a segment to 10 and the maximum number of segments to 35. This procedure partitioned the tracking records into segments that differed in their spatial locations. Expecting seasonal movements from the tracked Grey Herons population, we used changes in bird location to recognize the following phenological periods: wintering, breeding, spring migration, and autumn migration. For GER-NR, we distinguished an additional phase: the post-breeding dispersal period. Based on expert knowledge, we manually corrected single data records (*n* = 46) where outlier positions did not match the algorithm-derived segmentation pattern. Finally, Table 1 shows the start and end dates of each phase, alongside the duration of each phenological phase in days.

**Table 1:** The annual life cycle characteristics of the studied GPS-tracked Grey Herons (GER-NR, POL-KR and POL-OL). Described by: start and end of each phenological period and its duration (days), covered distance during migrations and movement within the post-breeding dispersal (km).

**Phenological phase**	**Start date**	**End date**	**Duration** **(days)**	**Movement** **distance (km)**
**GER-NR**				
wintering 1	05.11.2018	31.01.2019	88	–
spring migration 1	31.01.2019	02.02.2019	3	277.40
breeding 1	02.02.2019	26.05.2019	114	–
post-breeding dispersal 1	27.05.2019	30.09.2019	127	209.58
autumn migration 1	30.09.2019	02.10.2019	3	175.58
wintering 2	02.10.2019	27.01.2020	118	–
breeding 2	28.01.2020	21.04.2020	85	–
**POL-KR**				
breeding 1	12.05.2012	22.06.2012	41	–
autumn migration 1	05.07.2012	08.07.2012	4	1,133.07
stop-over 1	05.07.2012	07.07.2012	3	925.78
wintering 1	08.07.2012	05.12.2012	150	–
spring migration 1	05.03.2013	4-5.04.2013	32	1,116.33
breeding 2	28.04.2013	21.07.2013	85	–
autumn migration 2	22.07.2013	05.08.2013	15	768.53
stop-over 1	22.07.2013	23.07.2013	2	103.30
stop-over 2	24.07.2013	03.08.2013	11	124.14
stop-over 3	04.08.2013	04.08.2013	1	248.97
stop-over 4	05.08.2013	05.08.2013	1	148.05
wintering 2	06.08.2013	28.10.2013	84	–
**POL-OL**				
breeding 1	21.07.2015	27.08.2015	38	–
autumn migration 1	27.08.2015	04.09.2015	9	1,758.05
stop-over 1	27.08.2015	28.08.2015	1	35.35
stop-over 2	28.08.2015	29.08.2015	1	361.47
stop-over 3	30.08.2015	30.08.2015	1	198.02
stop-over 4	30.01.2015	31.08.2015	1	77.27
stop-over 5	31.08.2015	31.08.2015	1	171.96
stop-over 6	01.09.2015	01.09.2015	1	36.70
stop-over 7	01.09.2015	01.09.2015	1	389.14
stop-over 8	02.09.2015	02.09.2015	1	53.70
stop-over 9	03.09.2015	04.09.2015	2	165.78
wintering 1	05.09.2015	26.02.2016	175	–
spring migration 1	26.02.2016	02.03.2016	6	1,605.17
stop-over 1	29.02.2016	01.03.2016	2	717.93
breeding 2	02.03.2016	05.09.2016	188	–
autumn migration 2	05.09.2016	08.09.2016	3	1,622.16
stop-over 1	07.09.2016	08.09.2016	1	1,227.20
wintering 2	01.10.2016	23.02.2017	146	–
spring migration 2	24.02.2017	27.02.2017	4	1,621.96
stop-over 1	24.02.2027	25.02.2017	2	946.00
breeding 3	27.02.2017	28.08.2017	183	–
autumn migration 3	28.08.2017	02.09.2017	6	1,660.54
stop-over 1	29.08.2017	29.08.2017	1	397.77
stop-over 2	30.08.2017	30.08.2017	1	101.59
stop-over 3	31.08.2017	31.08.2017	1	15.48
wintering 3	03.09.2017	14.02.2018	165	–
spring migration 3	15.02.2018	05.03.2018	19	1,680.19
stop-over 1	16.02.2018	02.03.2018	15	189.70
stop-over 2	03.03.2018	03.03.2018	1	481.76
stop-over 3	03.03.2018	04.03.2018	2	9.21
stop-over 4	05.03.2018	05.03.2018	1	352.87
breeding 4	03.03.2018	05.06.2018	95	–

#### Characteristics of migration

We determined several flight characteristics for each individual. We defined spring and autumn migrations as the time frame between a bird’s last GPS location at a stationary phase (breeding/wintering) and the first location at its next stationary phase (wintering/breeding), separated by a period of fast directional movement. For each migration, we quantified the number and duration of stop-overs, the distance travelled between successive stop-overs, the total migration length (in days), and the total distance travelled (the sum of the distances between the start and end positions, including all stop-over sites) (Table 1). We defined a stop-over as a period of decreased movement during migration, indicated by at least 3 GPS fixes located within 1 km of each other, collected over the course of at least 24 h (Manikowska-Ślepowrońska et al., 2025).

To calculate the distances covered by individuals between breeding and wintering sites, as well as between subsequent stop-over sites, we determined the geographic centres (centroids; point features) for each stage of the annual avian cycle. For this purpose, we used the *Central Feature* tool from the spatial statistics toolbox in ArcGIS Pro software ver. 3.4 (ESRI, 2024). We then measured the distances (in km) between the centroids of specific sites using the measurement tools provided in ArcGIS Pro software ver. 3.4 (ESRI, 2024).

#### Distance from the release site

We calculated and visualized the distances covered from the tagging and release site (the first GPS record) for each individual to describe their year-round movement patterns. This approach allowed us to identify character of annual movement patterns and reveal individual migration strategies. Similar distances from the tagging and release site during a specific phenological stage would indicate the use of the same breeding or wintering grounds (philopatry). Conversely, varying distances during the same phenological stage would indicate the use of different sites. To calculate the distances between the first GPS record and subsequent GPS locations, we used the *distGeo* function from the *geosphere* package (Hijmans et al., 2017). We then visualized the data using the *ggplot2* package (Wickham, 2016).

#### Inter-population differences in Grey Heron

We performed the Analysis of Variance from Summary Data (Larson, 1992) (hereafter (ANOVA) to compare migratory parameters between Grey Heron populations from Europe (this study; Manikowska-Ślepowrońska, Mokwa & Jakubas, 2018) and Asia (Far East Russia Tiunov et al., 2022) and S Korea (Lim et al., 2021). To detect potential differences between age groups, we also compared data between adult or subadult individuals (this study; Manikowska-Ślepowrońska, Mokwa & Jakubas, 2018; Lim et al., 2021) and juveniles (Tiunov et al., 2022). We conducted the ANOVA on the reported means and standard deviations (SD) values of specific migratory parameters (distance and duration of single migrations, and the number and duration of stop-overs) from the mentioned studies. We selected data from these references because they provide information on more than one individual, thus ensuring more reliable findings. In the case of significant differences, we conducted a *post-hoc* Tukey Honestly Significant Difference test (Tukey HSD) to identify specific pairwise differences between the studies.

## Results

### The annual cycle

**Figure 2:**
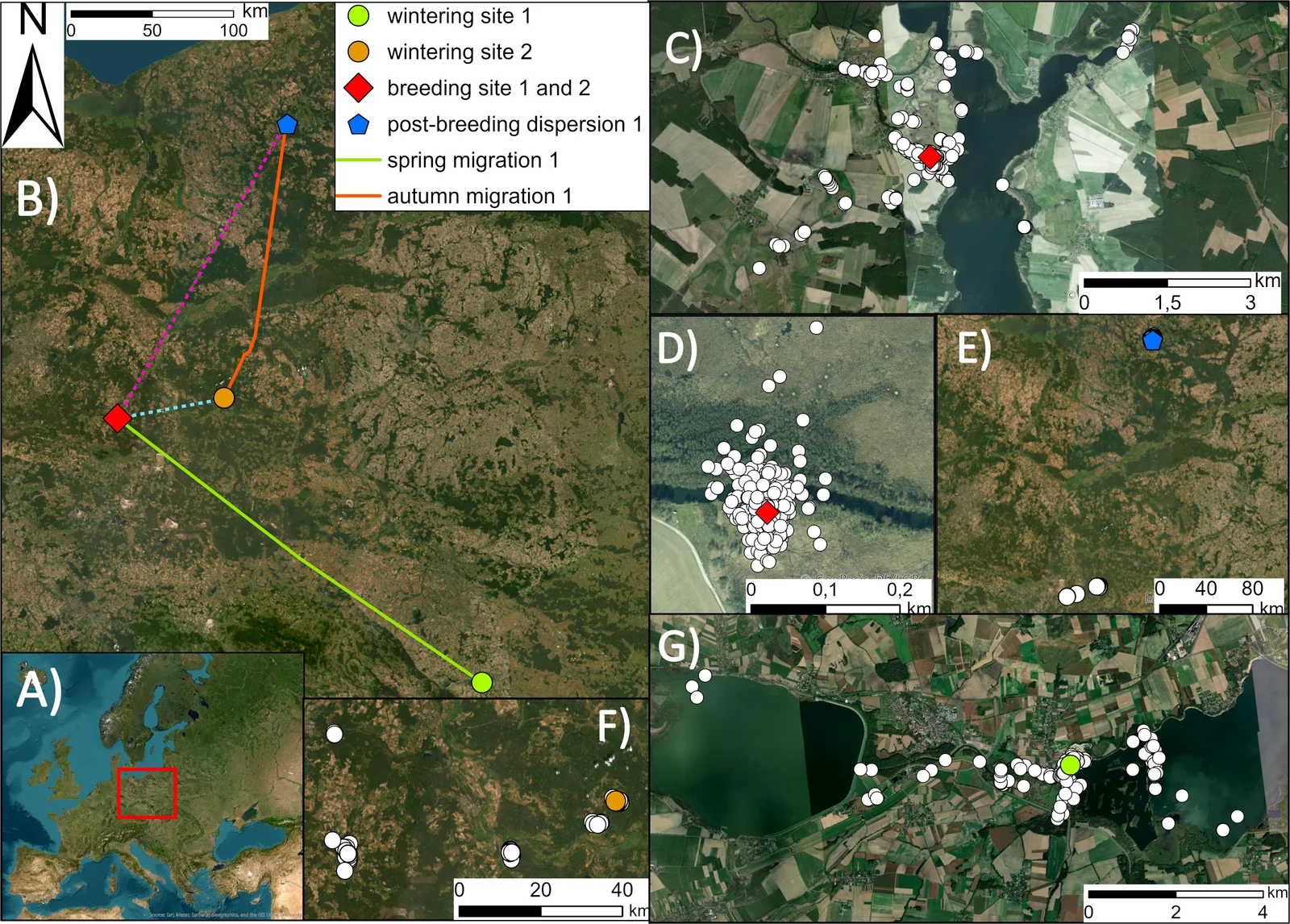
Movements of the GER-NR individual (caught and deployed with GPS logger in 1^st^ wintering site, S Poland). (A) Range of registered annual movements of the GER-NR individual on the continental scale. (B) all registered GPS tracks of the GER-NR individual (dashed lines –no GPS data, hypothetical direct flight line movement route, purple: between 1^st^ breeding and post-breeding dispersal, cyan: between 2nd wintering and 2^nd^ breeding). (C) breeding site in 2019. (D) breeding site in 2020. (E) post-breeding dispersal in 2019. (F) wintering site in 2018/2019. (G) wintering site in 2019/2020. Base map provided by Esri (2025). Coloured symbols indicate the geographic centre (centroid) of a particular site, white points show registered GPS locations in the particular phenological phases.

**Figure 3:**
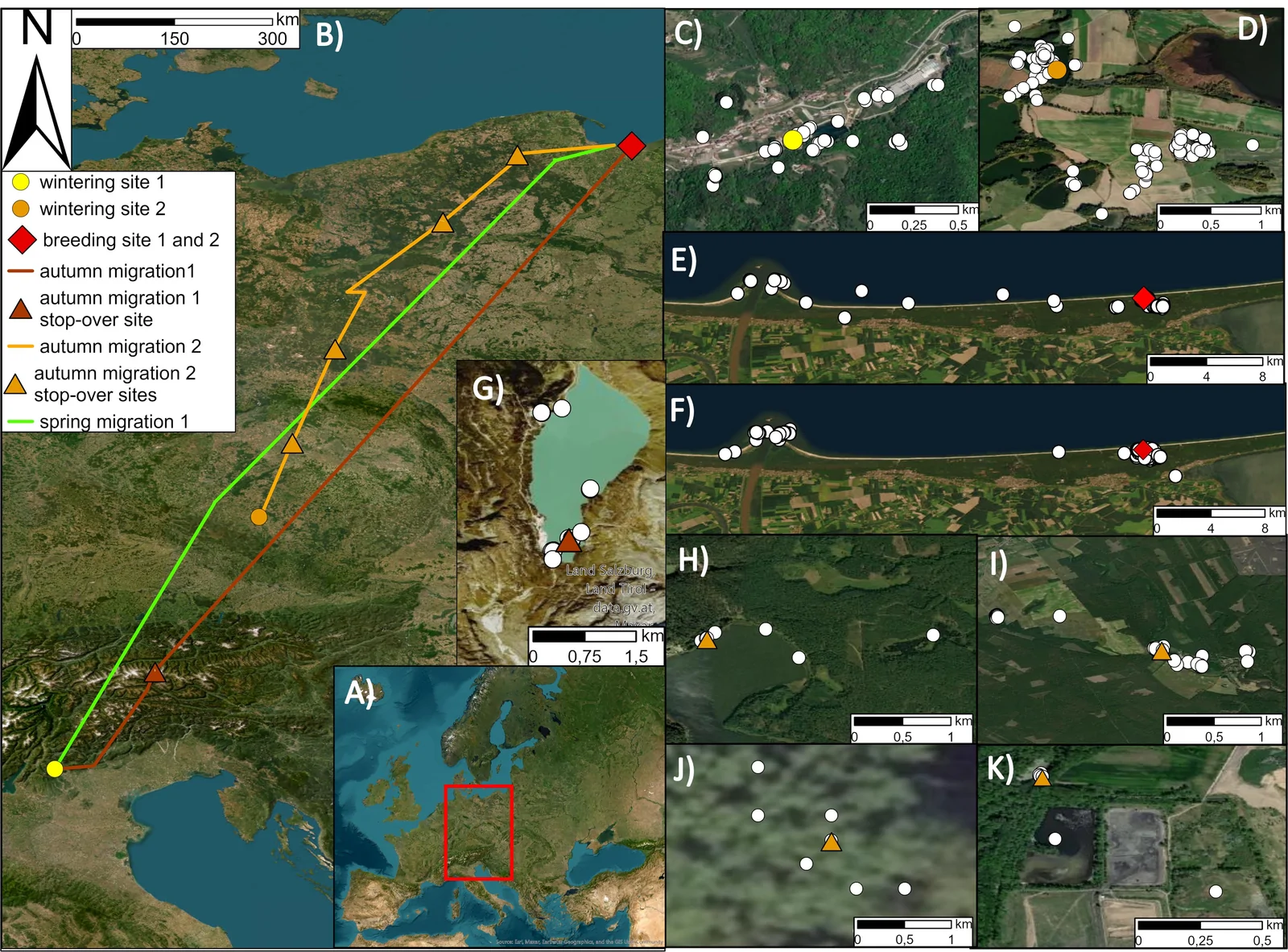
Movements of the POL-KR individual (caught and deployed with GPS logger in 1^st^ breeding site, N Poland). (A) Range of registered annual movements of the POL-KR individual on the continental scale. (B) whole GPS-tracked journey of the POL-KR individual. (C) wintering site in 2012–013. (D) wintering site in 2013–2014. (E) breeding site in 2012. (F) breeding site in 2013. (G) stop-over site during autumn migration in 2012. (H) 1^st^ stop-over site during autumn migration in 2013. (I) 2^nd^ stop-over site during autumn migration in 2013. (J) 3^rd^ stop-over site during autumn migration in 2013. (K) 4^th^ stop-over site during autumn migration in 2013. Base map provided by Esri (2025). Coloured symbols indicate the geographic centre (centroid) of a particular site, white points show registered GPS locations in the particular phenological phases.

**Figure 4:**
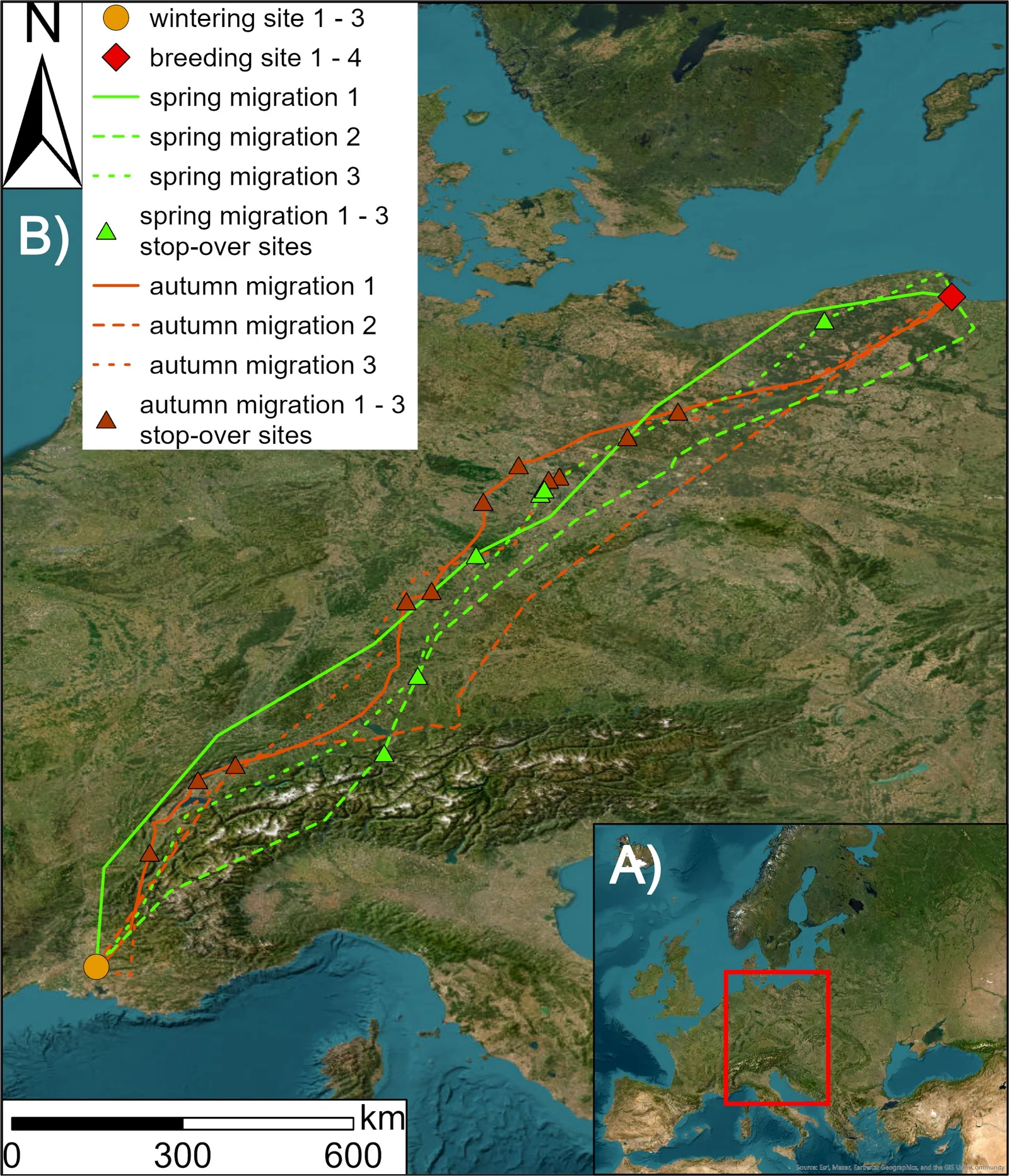
Movements of the POL-OL individual (caught and deployed with GPS logger in 1^st^ breeding site, N Poland). (A) Range of registered annual movements of the POL-OL individual on the continental scale. (B) whole GPS-tracked journey of the POL-OL individual. Base map provided by Esri (2025). Coloured symbols indicate the geographic centre (centroid) of the particular site, white points show registered GPS locations in the particular phenological phases.

We identified four main phases in the annual cycle of all studied individuals: breeding, wintering, spring migration, and autumn migration (Figs. 2–4; Table 1). Additionally, for GER-NR, we distinguished one more stage: post-breeding dispersal (Fig. 2; Table 1). This phase lasted 127 days, during which the bird travelled 209.6 km from its breeding colony in E Germany to the site where it spent this phenological period in NW Poland (Fig. 2; Table 1).

### Breeding and wintering grounds

Based on the GPS-tracking, we successfully identified the specific breeding locations of the studied individuals. GER-NR was breeding in a colony (estimated at 100 breeding pairs; Landesumweltamt Brandenburg, 2008) on the shore of Schwielochsee, a lake in Lower Lusatia (SE Brandenburg, E Germany). POL-OL was breeding on the shore of a small pond in the ZOO in Gdańsk (Gdańsk Costal Zone, N Poland) (colony size estimated at 29 pairs; Manikowska-Ślepowrońska et al., 2015). Lastly, POL-KR was breeding in a colony in the forest on the Vistula Spit, located close to the coast of the Gulf of Gdańsk and the Vistula Lagoon in N Poland (colony size estimated at 326 pairs in the studied years; Manikowska-Ślepowrońska et al., 2015).

**Figure 5:**
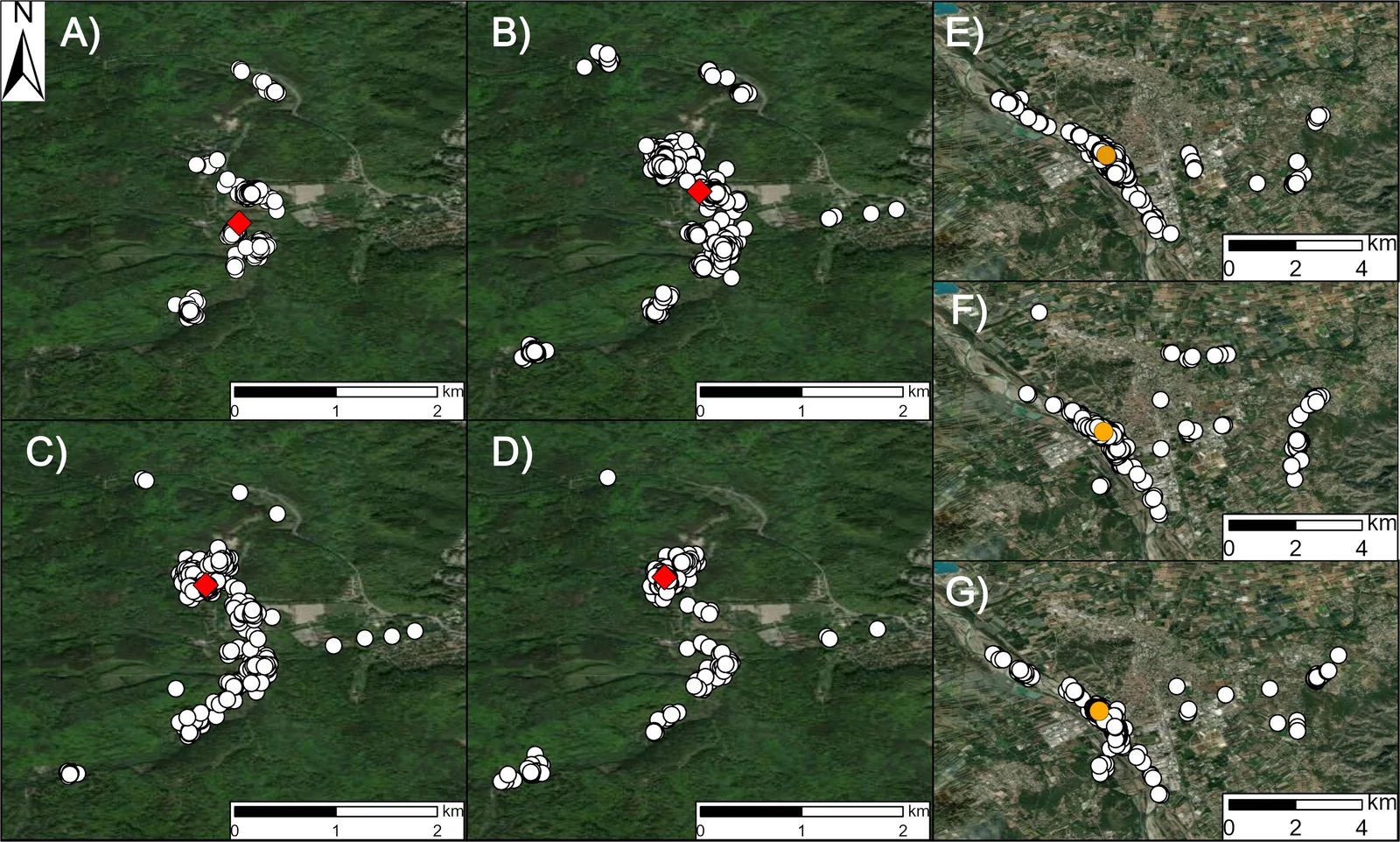
Detailed movements of the POL-OL individual. (A–D) Breeding sites 1^st^–4^th^ (2015 –2018). (E–G) wintering sites 1^st^–3^rd^ (2015/2016–2017/2018). Base map provided by Esri (2025). Coloured symbols indicate the geographic centre (centroid)–red squares for the breeding site, orange circles for wintering sites, white points show registered GPS positions in the particular phenological phases.

GER-NR and POL-KR were tracked during two breeding and two wintering seasons. Both birds bred in the same colony across consecutive seasons, but changed their wintering quarters from year to year. GER-NR spent its first winter in SW Poland at Lake Głębinowskie near the village of Wójcice. In the second winter, this bird stayed in W Poland, utilizing fish ponds and a river between/connecting Lake Kolno and Lake Środkowe in the village of Bytnica (Fig. 2). Similarly, POL-KR spent its first winter in NE Italy at Lake Posina near the small town of Posina, but it moved to S Czech Republic for its second winter, foraging on fish ponds (Fig. 3). Lastly, we tracked POL-OL throughout four breeding seasons in the same colony and three wintering seasons at the same wintering ground in S France, specifically near the bridge over the Durance River close to the centre of the middle-sized town of Cavaillon (Figs. 4–5).

The breeding period ranged from 85 to 188 days (mean ± SD: 143 ± 51 days); POL-KR registered the shortest breeding phase, whereas POL-OL exhibited the longest (Table 1). The tracked Grey Herons spent between 118 and 175 days on their wintering grounds (151 ± 22 days) (Figs. 2–5; Table 1). Among them, GER-NR stayed on the wintering grounds for the shortest period compared to POL-KR and POL-OL (Table 1).

### Migration patterns

**Figure 6:**
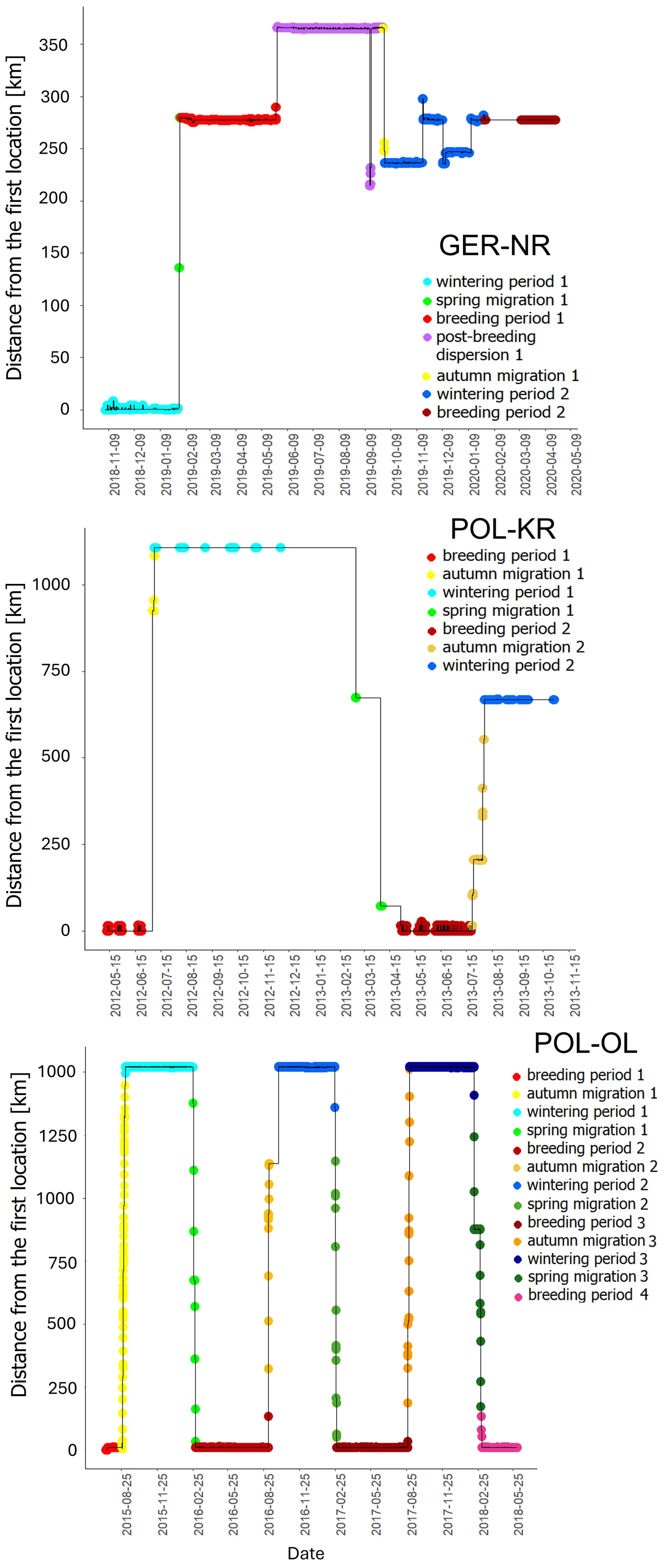
Distances between the particular GPS locations of GPS-tracked Grey Heron adult individuals to the logger deployment site (km) in particular dates. Assigned colour codes correspond to avian annual cycle phases (red tones–breeding periods; blue tones–wintering periods; green tones–spring migration periods; yellow-orange tones–autumn migration periods; violet –post-breeding dispersal period).

**Figure 7:**
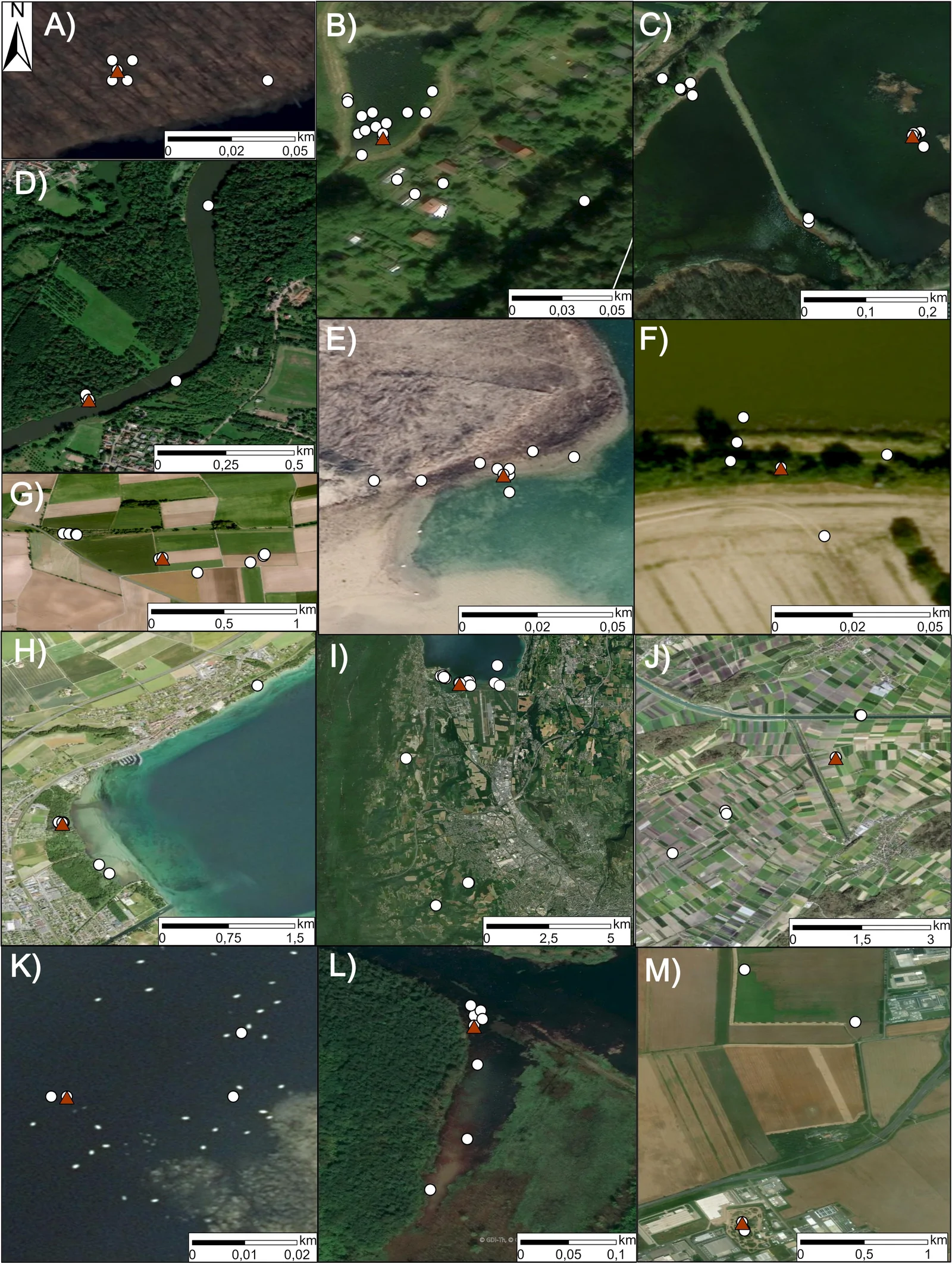
Stop-over sites during three autumn migrations of the POL-OL individual. (A–I) stop-over sites (*n* = 9) during 1^st^ autumn migration. (J) the only stop-over site during 2^nd^ autumn migration. (K–M) stop-over sites during 3^rd^ autumn migration (*n* = 3). Base map provided by (ESRI, 2024). Red triangle indicates geographic centre (centroid) of each stop-over site, white points show registered GPS locations at the particular stop-overs.

**Figure 8:**
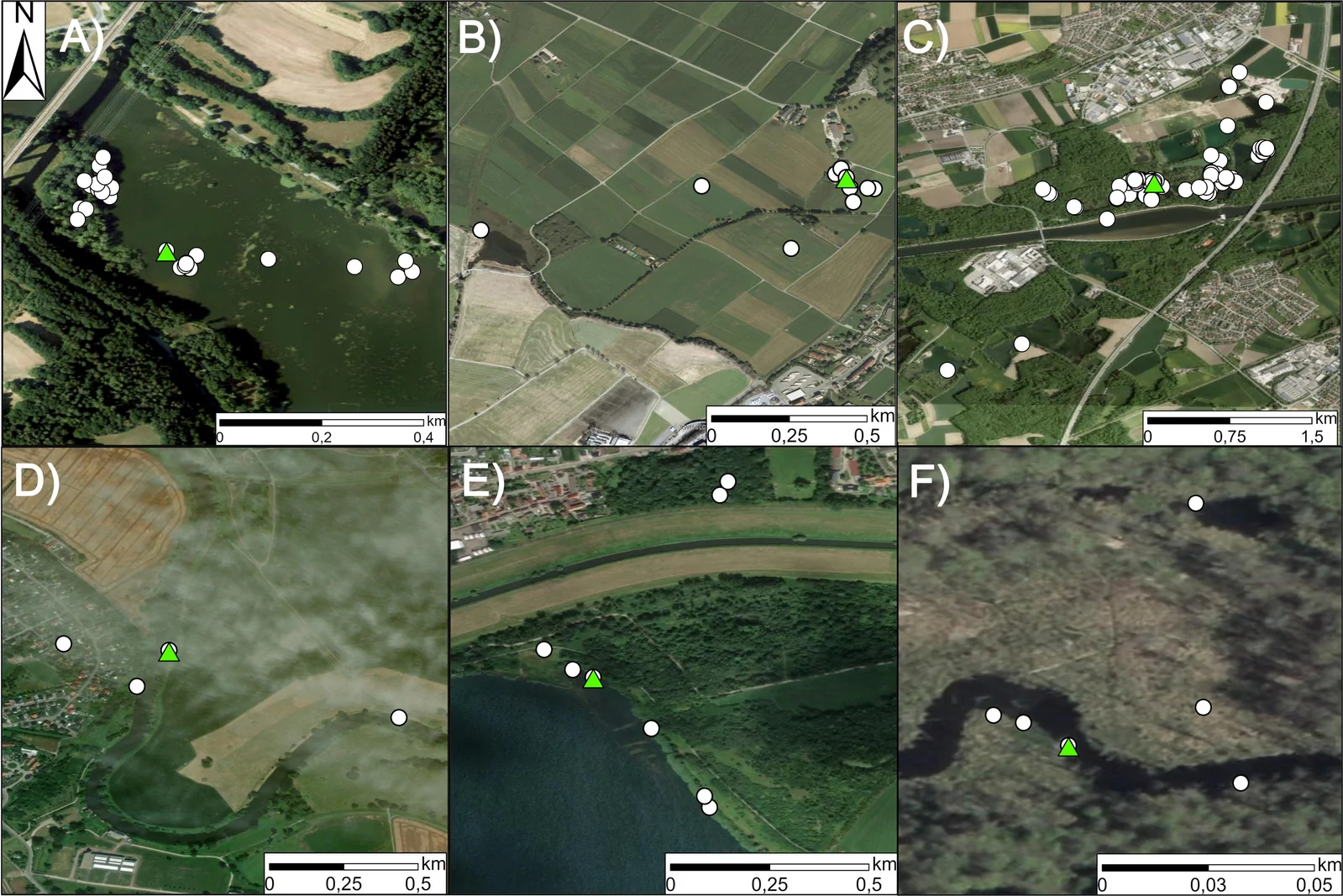
Stop-over sites during the three spring migrations of POL-OL individual. (A) the only stop-over site during 1^st^ spring migration. (B) the only stop-over during 2^nd^ spring migration. (C–F) stop-over sites (*n* = 4) during 3^rd^ spring migration. Base map provided by ESRI (2024). Green triangle indicates geographic centre (centroid) of each stop-over site, white points show registered GPS locations at the particular stop-overs.

Unlike the other tracked individuals, POL-OL followed a similar pattern of migration in subsequent years. When we visualized the distance travelled over time from the release point, we found a regular pattern with similar durations and distances (Fig. 6); this consistency applied to all three spring migrations and three autumn migrations (Table 1). In contrast, the migrations of GER-NR were noticeably shorter in duration, lasting the same number of days in both seasons. The migration durations of POL-KR varied between the spring and autumn migrations (Table 1). POL-OL regularly migrated to the same breeding and wintering sites (Figs. 4–8) at remarkably similar times each year (Fig. 5). Consequently, POL-OL travelled the longest cumulative distances during spring (mean ± SD: 1,635.8 ± 39.37 km) and autumn migrations (1,680.2 ± 70.06 km), whereas GER-NR covered the shortest distances (Table 1), and POL-KR exhibited intermediate values (Fig. 4; Table 1).

### Stop-over sites

We recorded stop-over sites during all spring and autumn migrations of POL-KR and POL-OL. For POL-KR, we recorded a single stop-over that lasted 3 days during its first autumn migration. During the second autumn migration of this individual, we distinguished four stop-over sites, with durations ranking from 1 to 11 days (mean ± SD: 4.33 ± 5.77 days; Table 1). In the case of POL-OL, we found that most stop-overs lasted for one or two days during both migrations. Only one stop-over, during its third spring migration, lasted as long as 15 days (Table 1). Lastly, we could not distinguish any stop-overs for GER-NR, which probably reflects its very short migration periods and the relatively short distances it covered.

### Summary of the Grey Heron migration

We found that the duration of autumn migration differed significantly between our study and other Grey Heron populations (ANOVA, *df* = 3, *F* = 56.31, *P* < 0.001). Specifically, juvenile Grey Herons from Far East Russia (Tiunov et al., 2022) performed longer autumn migrations compared to adult individuals from this study (Tukey HSD *post-hoc* test, 95% CI [1,210.0–2,321.2 km], *P* < 0.001) and adults individuals ringed in Poland (Manikowska-Ślepowrońska, Mokwa & Jakubas, 2018) (Tukey HSD *post-hoc* test, 95% CI [1,398.5–2,095.3 km], *P* < 0.001) (Table 2). Finally, adult Grey Herons ringed in Poland (Manikowska-Ślepowrońska, Mokwa & Jakubas, 2018) covered shorter distances during autumn migration compared to adult Grey Herons from South Korea (Lim et al., 2021) (Tukey HSD *post-hoc* test, 95% CI [−2,393.6 to −726.9 km], *P* < 0.001) (Table 2).

**Table 2:** Summary of selected data on migration of Grey Heron. Values for studies with multiple individuals are reported as means (mean ± SD). AM, autumn migration; SM, spring migration; AMS, autumn migration stop-over; SMS, spring migration stop-over; n.d., no data.

**Variable**	**Data source**					
	**This study**	**Manikowska-Ślepowrońska, Mokwa & Jakubas, 2018**	**Ye et al., 2018**	**Tiunov et al., 2022**	**Lim et al., 2021**	**Kim et al., 2015**
Region	Central Europe	Poland	South East China	Far East Russia	South Korea	South Korea
Approach	GPS/GSM	Ring recoveries	GPS/GSM	GPS/GSM	GPS/GSM	GPS/GSM
Age of birds (No. of individuals; Effective sample size)	adult (3; 6)	adults and subadults (206; 206)	adult (1;2)	juvenile (8;10)	adult (4;2)	juvenile (1;1)
AM duration (days)	6.7 ± 4.7	n.d.	5.7	10.1 ± 11.2	8.0 ± 5.7	41
SM duration (days)	12.8 ± 12.5	n.d.	4.1 ± 2.1	19.4 ± 15.1	n.d.	n.d.
AM distance (km)	1,186.3 ± 623.0	1,205 ± 224.9	3,003.0	2,951.9 ± 1,685.0	1,391.7 ± 392.5	730
SM distance (km)	1,260.2 ± 594.3	n.d.	2,781.5 ± 99.7	3,187.0 ± 1,498.0	n.d.	n.d.
AM pace (km/day)	177.0	n.d.	525.9	292.3	174.0	n.d.
SM pace (km/day)	46.4	n.d.	678.4	164.3	n.d.	n.d.
Time of the day	Day & Night	n.d.	Day & Night	Mostly night	n.d.	n.d.
Number of AMS	3.6 ± 3.29	n.d.	0.0	8.0 ± 1.6	8.0 ± 2.8	1.0
Number of SMS	2.0 ± 1.73	n.d.	n.d.	n.d.	n.d.	n.d.
Duration of AMS (days)	1.8 ± 2.37	n.d.	0.0	2.3 ± 7.4	n.d.	39
Duration of SMS (days)	3.8 ± 5.49	n.d.	n.d.	n.d.	n.d.	n.d.

We found that the number of stop-overs during autumn migration differed between Grey Heron populations (ANOVA, *df* = 2, *F* = 6.75, *P* = 0.01) with individuals from Far East Russia (Tiunov et al., 2022) making more stop-overs compared to individuals from this study (Tukey HSD *post-hoc* test, 95% CI [1.2–7.6], *P* = 0.01) (Table 2).

The duration of autumn migration remained similar among Grey Heron populations from this study, Far East Russia (Tiunov et al., 2022), and South Korea (Lim et al., 2021) (ANOVA, *df* = 2, *F* = 0.26, *P* = 0.77). Similarly, the duration of autumn stop-overs did not differ between our study and Grey Heron population investigated in Far East Russia (Tiunov et al., 2022) (ANOVA, *df* = 1, *F* = 0.02, *P* = 0.88).

Juveniles from Far East Russia (Tiunov et al., 2022) travelled further than individuals from this study during spring migration (ANOVA, *df* = 1, *F* = 7.45, *P* = 0.02) (Table 2). However, the duration of spring migration remained similar between Grey Heron populations from this study and young herons from Far East Russia (Tiunov et al., 2022) (ANOVA, *df* = 1, *F* = 0.70, *P* = 0.42).

## Discussion

### Breeding and wintering grounds

All studied individuals returned to the same breeding sites in consecutive years. Herons and egrets typically exhibit high fidelity to their breeding colonies (Van Vessem & Draulans, 1987; Bancroft, Ogden & Patty, 1988; Van der Winden, Poot & Van Horssen, 2010; Carrasco, Toquenaga & Mashiko, 2017; Ye et al., 2018; Huang et al., 2022).

The literature indicates that there is still relatively little known about the non-breeding site fidelity of herons and egrets (Koczur et al., 2018; McCrimmon Jr et al., 2020). POL-OL utilized the same wintering site in three consecutive seasons, while POL-KR and GER-NR changed their wintering grounds every year. In other studies, some Grey Heron (Ye et al., 2018; Tiunov et al., 2022) and Great Egret (Lumpkin et al., 2023) individuals also spent the winter in the same areas in consecutive years. GER-NR and POL-KR spent their second winter (following logger deployment) closer to the breeding site compared to their first winter. The literature reports that this phenomenon of decreasing the distance to the wintering ground in successive winters also occurs in the Great Egret (Lumpkin et al., 2023). It is common for birds to decrease their migration distances with age, as documented in the Grey Heron (Wernham et al., 2002; Bakken, Runde & Tjřrve, 2003), the Great Egret (Włodarczyk et al., 2020), and other avian species (Newton, 2010). While warmer winters due to climate change have been suggested as a broader cause of birds wintering closer to their breeding colonies more often due to unfrozen foraging grounds (Newton, 2010), our small sample size does not allow us to determine whether the observed shifts reflect such large-scale environmental changes, age-dependent experience, or simply individual variation.

### Migration patterns

All studied GPS-tracked individuals exhibited a distinctive movement pattern. The individuals breeding in N Poland performed relatively long-distance migrations (max. distance between the breeding and wintering sites: 768–1,758 km) along a SW–NE direction. In contrast, the individual breeding in E Germany moved relatively short distances from its colony; it travelled 210 km northward during post-breeding dispersal and subsequently moved another 176 km to a wintering site located close to its breeding grounds (in the eastern area). The observed differences in the GER-NR’s translocation pattern could be due to its rehabilitation period. Nevertheless, the observed distance patterns are consistent with the movement properties derived from the literature. Post-breeding dispersal in the close proximity of the colony has been also observed in other heron and egret species (Van Vessem & Draulans, 1987; Fransson & Pettersson, 2001; Fasola et al., 2002; Van der Winden, Poot & Van Horssen, 2010; Manikowska-Ślepowrońska, Mokwa & Jakubas, 2021).

The migration directions and wintering ground locations observed in this study are highly consistent with ring recovery data for this species in Central Europe (Rydzewski, 1956; Manikowska-Ślepowrońska, Mokwa & Jakubas, 2018; Manikowska-Ślepowrońska, Mokwa & Jakubas, 2021) and with other studies on European populations (Cramp, 1998; Bakken, Runde & Tjřrve, 2003; Bønløkke et al., 2006; Cepák et al., 2008). The individual nesting in E Germany spent the non-breeding periods, including the wintering phases, relatively close to its breeding site. Its compromised health status (having been released after treatment in a wildlife asylum) may have restricted its migration range. On the other hand, its movement distances during post-breeding dispersal and wintering align with the know pattern of decreasing migration distance in Grey Herons with decreasing breeding latitude (Manikowska-Ślepowrońska, Mokwa & Jakubas, 2018). Indeed, differences in the annual movement pattern of the GER-NR, compared to the two other herons from N Poland, could potentially reflect individual variations or tentative effects of a latitudinal gradient among the breeding areas, as suggested by (Manikowska-Ślepowrońska, Mokwa & Jakubas, 2021).

The spring and autumn migrations of all tracked individuals lasted between 3 and 32 days (Table 1). These durations align with previous studies on *Ardeidae*, including the Grey Heron, Chinese Egret, Eurasian Bittern, and Purple Heron (see Supplementary Materials Table S1). The studied birds started their autumn migrations between 5 July (POL-KR) and 30 September (GER-NR). This timeframe begins slightly earlier than the early September to late October window generally reported in the literature (Cramp, 1998). Although the GER-NR overwintered closest to its breeding ground, it was the last to depart on autumn migration (30 September). Overall, the migration timing broadly matches the dates previously reported for both Grey Heron (Tiunov et al., 2022) and Purple Heron (van der Winden, Poot & Van Horssen, 2010). The individuals initiated their return to the breeding sites in February, consistently with literature records (Cramp, 1998). The only exceptions were GER-NR, which started its spring migration at the final day of January, and POL-KR, which departed at the beginning of March (Table 1).

Our results indicate that herons can depart from the colony shortly after completing the breeding season, though some individuals delay departure until late autumn. Several factors may influence the timing of departure from breeding grounds, including migration distance, environmental conditions in the immediate proximity of the colony, breeding failure, or individual body condition.

Our findings demonstrate that the individual overwintering closest to its breeding ground in E Germany initiated the breeding season earliest, specifically between late January and early February. This individual also spent the least amount of time at its wintering site. In contrast, the wintering grounds of the other two individuals were located further from their respective breeding colonies; consequently, they arrived at the colonies later, specifically between late February and early March (POL-OL) or in late April (POL-KR). Among all surveyed individuals, POL-OL, which bred in N Poland, spent the greatest number of days at its wintering site. Our results align with the known breeding phenology of this species in Europe (Milstein, Prestt & Bell, 1970; Van Vessem & Draulans, 1987; Cramp, 1998; Jakubas, 2011). Furthermore, this pattern of breeding onset relative to the distance from wintering quarters matches observations of Great Egrets in California, USA, where GPS-tracked individuals wintering furthest from the colony arrived latest at the breeding sites (Lumpkin et al., 2023). More broadly, birds that winter closer to their breeding grounds tend to arrive earlier in spring and depart later in autumn than those wintering further away (Newton, 2010).

### Stop-over sites

As expected, the studied individuals breeding in N Poland adopted an intermittent migration strategy, utilizing several stop-over sites where they stayed for an average of 2.3 ± 3.4 days. Previous research documented a similar stop-over duration for the Chinese Egret during autumn migration (approx. three to approx. seven days; (Xu et al., 2022). Likewise, other studies have reported intermittent migration strategies with multiple stop-over sites for Grey Heron (Kim et al., 2015; Lim et al., 2021), Chinese Egret (Huang et al., 2022) and Reddish Egret (Koczur et al., 2018). In the Night Heron, researchers have also observed short diurnal stop-overs between nocturnal migratory flights (Ledwoń & Betleja, 2014). Furthermore, Grey Herons breeding in Far East Russia have exhibited diverse migration patterns, ranging from long, rapid flights with few stop-overs to journeys with stop-overs lasting several weeks (Tiunov et al., 2022).

In contrast, the individual breeding in E Germany performed short migrations without any detectable stop-overs. This absence could stem from gaps in GPS recording or from its post-breeding dispersal behaviour, which we recorded exclusively for this individual. Given the short migration distance, this bird likely did not require stop-overs. Migration route lacking stop-overs has already been documented for the GPS-tracked Grey Herons from China (Ye et al., 2018). Similarly, it has been claimed that the Purple Heron migrates to its wintering grounds without long stop-over *en route* (Van der Winden, Poot & Van Horssen, 2010). However, authors detected shorter diurnal stops-overs. Those authors also attributed the absence of longer recorded stop-overs to battery limitations and gaps in the GPS signal.

The stop-over durations for POL-OL ranged from one to 15 days; most usually lasted a single day (15 cases), while the remaining five stop-overs lasted between two and 15 days. For POL-KR, stop-overs lasted between one and 11 days. Considering the habitat types that these GPS-tracked individuals exploited during stop-overs (*e.g.*, fish ponds and small lakes), the birds highly likely refuelled effectively before resuming their migration.

### Summary of Grey Heron migration

Regarding migration distances, our revised inter-population analysis confirmed that the Grey Heron exhibits highly flexible migratory, semi-migratory, and dispersive behaviours across Palearctic populations. The autumn migration distance differed significantly among the compared Eurasian populations, revealing distinct geographic and age-related patterns reflecting population adaptation to local environmental conditions. Generally, literature indicates that Grey Heron populations from northern latitudes migrate further and more frequently than those from lower latitudes, due to harsher winter conditions and the freezing of foraging grounds (Voisin, 1991; Manikowska-Ślepowrońska, Mokwa & Jakubas, 2018). Our statistical analysis reflects this latitudinal gradient, as Grey Herons from Poland covered shorter autumn distances compared to adult Grey Herons from South Korea (Lim et al., 2021), while also aligning with previous European ringing recoveries showing slightly longer migration distances for northern Polish populations than for those from southern Poland (Rydzewski, 1956; Manikowska-Ślepowrońska, Mokwa & Jakubas, 2018) (Table 2). Furthermore, we found age-related differences within these geographic patterns. Juvenile herons from Far East Russia (Tiunov et al., 2022) performed longer autumn and spring migrations than both the adult individuals from our study and the adult/subadult birds ringed in Poland (Manikowska-Ślepowrońska, Mokwa & Jakubas, 2018). This contrasts with Western and Central European populations, which typically show a higher prevalence of shorter, non-migratory movements or localized dispersal (Voisin, 1991; Cramp, 1998).

Despite these distinct spatial differences, the temporal characteristics and stop-over strategies showed consistency across regions. Analyses suggest that individuals from all studied populations performed intermittent flights, utilizing a few stop-overs that ranged in duration from one to 39 days (this study; Kim et al., 2015). However, the specific number of autumn stop-overs varied between populations; the herons tracked in our study made fewer stop-overs than the juveniles from Far East Russia (Tiunov et al., 2022). Interestingly, the overall duration of both autumn and spring migrations, as well as the duration of autumn stop-overs, remained statistically similar between our study and the studies on the Asian populations (Lim et al., 2021; Tiunov et al., 2022). This suggests that while migration distances and stop-over frequencies vary widely based on age and geographic origin, the overall time investment required for migration remains relatively uniform across these Palearctic populations.

We are aware of certain limitations of the analyses conducted and interpreting the results from the ANOVA from summary data, as this type of analysis may be biased by the inability to verify underlying assumptions on the data such as normality and homogeneity of variances; also, the Tukey test may be sensitive to differences in sample sizes between groups.

### Limitation of the study

Our study has some limitations. First, we based this research on only three adult individuals of Grey Herons. Undoubtedly, a limited dataset from a few locations provides little insight into the variability of migratory strategies within the species. For this reason, we recommend future studies including more individuals caught at a wider spatial scale. Second, we encountered some gaps in the GPS-tracking data, which did not allow us to continuously track individuals during certain periods. Additionally, the method of logger attachment might have affected bird behaviour and, in turn, yielded results differing from the natural behaviour present in unburdened individuals. This could have an especially strong impact on the rehabilitated individual. Nevertheless our study provides the first description of the annual movements of Central European Grey Herons and a valuable insight into their migration strategy.

## Conclusions

The annual movement pattern of the Grey Heron is variable at an individual level. This variability includes the range of covered distances annually and fidelity to the areas used as breeding or wintering grounds. The studied individuals returned to the same colonies, but not all remained faithful to the same wintering grounds. The individual breeding in E Germany exhibited a rather short-distance migration compared to the other two studied individuals breeding in N Poland; this is consistent with the latitudinal migration patterns observed in other studies for ringing recovery data in Central Europe. The migration characteristics (duration and distance) of the studied Central European population are generally similar to those reported for Asian populations. Herons travel shorter routes during spring migrations than when migrating after the breeding season. Furthermore, the individuals from our study utilized stop-overs of shorter duration than those reported for Asian populations, which likely stems from a shorter migration after the breeding period. At a broader Eurasian scale, our comparisons revealed distinct age-related and geographical variations, with juveniles from Far East Russia performing longer migrations and making more stopovers during autumn compared to European adults. However, despite these spatial differences, the overall migration duration and stop-over durations remained uniform across the studied Palearctic populations, indicating a consistent temporal investment in migration regardless of age or geographic origin.

## Supplemental Information

10.7717/peerj.21659/supp-1 Supplemental Information 1 Raw Data for Manuscript

10.7717/peerj.21659/supp-2 Supplemental Information 2 Summary of selected morphological traits, and migratory characteristics in *Ardeidae*. Values are reported as mean ± SD if literature provides several records for the same index. * - information on distance based on the maximal distance from the natal colony. Mean wingspan (m), maximum range between both wingtips; AM, autumn migration; SM, spring migration; Age (ad., adult; juv., juvenile); n.d., no data.

## Additional Information and Declarations

### Field Study Permissions

The following information was supplied relating to field study approvals (i.e., approving body and any reference numbers): No specific permits were required for the described field studies. We added this sentence to Methods in manuscript. Research which was conducted at the Oliwa Zoological Garden in Gdańsk, Poland was verbal permission for capture and tagging activities on-site was granted by the Manager/Director of the Zoological Garden. I didn’t need any permits for that. If you have a bird banding license in Poland, you don’t need any permits to catch birds outside of private property—the license from the Minister (issued annually) allows you to do so. In the acknowledgments section, we expressed our gratitude to the zoo director.

### Data Availability

The following information was supplied regarding data availability: The raw data is available in the Supplemental Files.

## References

[ref-1] Alerstam T (2001). Detours in bird migration. Journal of Theoretical Biology.

[ref-2] Bakken V, Runde O, Tjřrve E (2003). Norsk ringmerkingsatlas. Vol. 1.

[ref-3] Bancroft GT, Ogden JC, Patty BW (1988). Wading bird colony formation and turnover relative to rainfall in the Corkscrew Swamp area of Florida during 1982 through 1985. Wilson Bulletin.

[ref-4] Bønløkke J, Madsen JJ, Thorup K, Pedersen KT, Bjerrum M, Rahbek C (2006). Dansk Trækfugleatlas.

[ref-5] Carrasco L, Toquenaga Y, Mashiko M (2017). Balance between site fidelity and habitat preferences in colony site selection by herons and egrets. Journal of Avian Biology.

[ref-6] Cepák J, Klvaňa P, Škopek J, Schröpfer L, Jelínek M, Hořák D, Formánek J, Zárybnický J (2008). Atlas migrace ptáků České republiky a Slovenska.

[ref-7] Cramp S (1998). The complete birds of the Western Palearctic. CD-ROM.

[ref-8] Dingle H, Drake VA (2007). What is migration?. Bioscience.

[ref-9] ESRI (2024). ArcGIS Pro: release 10.

[ref-10] Esri (2025). World Imagery [basemap] February 19 2012. http://www.arcgis.com/home/item.html?id30e5fe3149c34df1ba922e6f5bbf808f.

[ref-11] Fasola M, Hafner H, Kayser Y, Bennetts RE, Cezilly F (2002). Individual dispersal among colonies of little egrets *Egretta garzetta*. Ibis.

[ref-12] Finlayson C (1992). Birds of the Strait of Gibraltar.

[ref-13] Finlayson C (2010). Birds of the Strait of Gibraltar.

[ref-14] Fransson T, Pettersson J (2001). Swedish bird ringing atlas.

[ref-15] Gu D, Chai Y, Gu Y, Hou J, Cao L, Fox AD (2019). Annual migration routes, stopover patterns and diurnal activity of Eurasian Bitterns *Botaurus stellaris* wintering in China. Bird Study.

[ref-16] Hancock J, Kushlan JA (2010). The herons handbook.

[ref-17] Higuchi H, Pierre JP (2005). Satellite tracking and avian conservation in Asia. Landscape and Ecological Engineering.

[ref-18] Hijmans RJ, Karney C, Williams E, Vennes C, Hijmans MRJ (2017). Package ‘geosphere’. Spherical Trigonometry.

[ref-19] Huang Z, Zhou X, Fang W, Chen X (2022). Migration and wintering of vulnerable adult Chinese Egrets (*Egretta eulophotes*) revealed by GPS tracking. Avian Research.

[ref-20] Jakubas D (2011). The influence of climate conditions on breeding phenology of the Grey Heron *Ardea cinerea* L. in northern Poland. Polish Journal of Ecology.

[ref-21] Kang JH, Kim IK, Lee KS, Kwon IK, Lee H, Rhim SJ (2016). Home range and movement of juvenile black-faced spoonbill *Platalea minor* in South Korea. Journal of Ecology and Environment.

[ref-22] Kenward RE (2001). A manual for wildlife radio tagging.

[ref-23] Kim DW, Kim HJ, Kwon IK, Hwang JW, Kim JH (2015). Post-breeding dispersal and autumn migration of a juvenile Grey Heron (*Ardea cinerea*). The Korean Journal of Ornithology.

[ref-24] Koczur LM, Kent GM, Ballard BM, Meyer KD, Green MC (2018). Space use and movements of adult Reddish Egrets (*Egretta rufescens*) during winter. Waterbirds.

[ref-25] Landesumweltamt Brandenburg xx (2008). Schwielochsee und Alte Spreemündung. Ökologische Charakterisierung der wichtigsten Brutgebiete für Wasservögel in Brandenburg.

[ref-26] Larson DA (1992). Analysis of variance with just summary statistics as input. American Statistical Association.

[ref-27] Lavielle M (2005). Using penalized contrasts for the change-point problem. Signal Processing.

[ref-28] Ledwoń M, Betleja J (2014). Post-breeding migration of Night Herons *Nycticorax nycticorax* tracked by GPS/GSM transmitters. Journal of Ornithology.

[ref-29] Lim EH, Shin MS, Cho HJ, Kim IK, Shin YU, Oh HS, Lee EJ (2021). Migration and Home Range of the Grey Heron (*Ardea cinerea*) in the Republic of Korea. Waterbirds.

[ref-30] Liu G, Peng W, Wu Q, Xie P, Ciu X, Li X (2020). Satellite tracking of the natal dispersion of grey heron chicks in Taihu National Wetland Park. Advances in Environmental Protection.

[ref-31] Lumpkin D, Jennings S, Warnock N, Condeso TE (2023). Partial migration by great egrets *Ardea alba* in Coastal California. Waterbirds.

[ref-32] Manikowska-Ślepowrońska B, Cieślińska K, Ślepowroński K, Siekiera J, Goc M, Iliszko LM, Jakubas D (2025). Habitat selectivity during the annual life cycle of the grey Heron *Ardea cinerea* revealed by GPS-tracking. Anim Biotelemetry.

[ref-33] Manikowska-Ślepowrońska B, Lazarus M, Zółkoś K, Jakubas D (2015). Determinants of the re-occupation and size of Grey Heron *Ardea cinerea* breeding colonies in northern Poland. Ecological Research.

[ref-34] Manikowska-Ślepowrońska B, Mokwa T, Jakubas D (2018). Wintering and stop-over areas of grey herons (*Ardea cinerea*) breeding in central europe: a ring-recovery analysis. Annales Zoologici Fennici.

[ref-35] Manikowska-Ślepowrońska B, Mokwa T, Jakubas D (2021). Dispersal from the natal colony of the Grey Heron *Ardea cinerea* nesting in Poland. Acta Ornithologica.

[ref-36] Manikowska-Ślepowrońska B, Szydzik B, Jakubas D (2016). Determinants of the presence of conflict bird and mammal species at pond fisheries in western Poland. Aquatic Ecology.

[ref-37] Marion L (1989). Territorial feeding and colonial breeding are not mutually exclusive: the case of the Grey Heron (*Ardea cinerea*). Journal of Animal Ecology.

[ref-38] McCrimmon Jr DA, Ogden JC, Bancroft GT, Martinez-Vilalta A, Motis A, Kirwan GM, Boesman PFD (2020). Great Egret (*Ardea alba*), version 1.0. in Birds of the World.

[ref-39] Milstein PL, Prestt I, Bell AA (1970). The breeding cycle of the Grey Heron. Ardea.

[ref-40] Morganti M, Viganò E, Berlusconi A, Cioccarelli S, Fasola M (2021). Post-fledging habitat selection of a Purple Heron *Ardea purpurea* revealed by GPS/GSM telemetry. Avocetta.

[ref-41] Newton I (2010). The migration ecology of birds.

[ref-42] Patin R, Etienne MP, Lebarbier E, Chamaillé-Jammes S, Benhamou S (2018). Identifying stationary phases in multivariate time series for highlighting behavioural modes and home range settlements. Journal of Animal Ecology.

[ref-43] R Core Team (2022). R: a language and environment for statistical computing. https://www.R-project.org/.

[ref-44] Rydzewski W (1956). The nomadic movements and migration of the European Common Heron. Ardea.

[ref-45] Suchantke A (1960). Autumn heron migration on the Camargue coast. The birdlife.

[ref-46] Tiunov I, Katin I, Lee KN, Hong SK, Lee I, Yoon H, Lee E, Lee S (2022). Migration of Grey Heron from the Peter the Great Bay and the potential transmission routes of avian influenza. Journal of Asia-Pacific Biodiversity.

[ref-47] Van der Winden J, Poot MJM, Van Horssen PW (2010). Large birds can migrate fast: the post-breeding flight of the Purple Heron *Ardea purpurea* to the Sahel. Ardea.

[ref-48] Van der Winden J, Van Horssen PW, Poot MJ, Gyimesi A (2012). Pre-migratory behaviour of the Purple Heron in the Netherlands. Ardeola.

[ref-49] Van Vessem J, Draulans D (1987). Spatial distribution and time budget of radio-tagged grey herons. Ardea cinerea, during the breeding season. Journal of Zoology.

[ref-50] Voisin C (1991). The herons of Europe.

[ref-51] Wernham C, Toms M, Marchant J, Clark J, Siriwardena G, Baillie S (2002). The migration atlas: movements of birds of Britain and Ireland.

[ref-52] Wickham H (2016). Getting Started with ggplot2. ggplot2: elegant graphics for data analysis.

[ref-53] Włodarczyk R, Szafara D, Kaczmarek K, Janiszewski T, Minias P (2020). Migratory behaviour and survival of Great Egrets after range expansion in Central Europe. PeerJ.

[ref-54] Xu H, Yang Z, Liu D, Jia R, Chen L, Liang B, Zhang Z, Zhang G (2022). Autumn migration routes of fledgling Chinese Egrets (*Egretta eulophotes*) in Northeast China and their implications for conservation. Avian Research.

[ref-55] Ye X, Xum Z, Aharon-Rotman Y, Yu H, Cao L (2018). First description of Grey Heron *Ardea cinerea* migration recorded by GPS/GSM transmitter. Ornithological Science.

