# Supplementary material for: Individual year-round movement patterns of the GPS-tracked Grey Herons (Ardea cinerea) in Central Europe: a multi-year case study
Source: PeerJ. 2026 Sep 4;14:e21659. doi: 10.7717/peerj.21659 (PMC13549148; doi:10.7717/peerj.21659)
Supplement: Supplementary file 1 [file peerj-14-21659-s002.docx]

**Supplementary Materials for**

**Individual year-round movement patterns of the GPS-tracked Grey Herons (*Ardea cinerea*) in Central Europe: A multi-year case study**

Brygida Manikowska-Ślepowrońska^1^, Karolina Cieślińska^1^, Krzysztof Ślepowroński^1^, Ewa Poślińska^2^, Joachim Siekiera^3^, Michał Goc^1^, Lech M. Iliszko^1^, Dariusz Jakubas^1^

^1^ Department of Vertebrate Ecology and Zoology, Faculty of Biology, University of Gdańsk, ul. Wita Stwosza 59, 80-308 Gdańsk, Poland

^2^ Regional Directorate for Environmental Protection in Opole, ul. Firmowa 1, 45-594 Opole, Poland

^3^ Rzeczna 17, 47-300 Żywocice, Poland

Corresponding Author:

Brygida Manikowska-Ślepowrońska^1^

Department of Vertebrate Ecology and Zoology, Faculty of Biology, University of Gdańsk, ul. Wita Stwosza 59, 80-308 Gdańsk, Poland

Email address: biobms@ug.edu.pl

**Table S1.** Summary of selected morphological traits, and migratory characteristics in *Ardeidae*. Values are reported as mean ± SD if literature provides several records for the same index. * - information on distance based on the maximal distance from the natal colony. Mean wingspan [m] – maximum range between both wingtips; AM – autumn migration; SM – spring migration; Age (ad. – adult, juv. - juvenile); n.d. – no data.

| **Species** | **Agami Heron**  ***Agamia agami*** | **Cattle Egret**  ***Bubulcus ibis*** | **Chinese Egret**  ***Egretta eulophotes*** | **Great Egret**  ***Ardea alba*** | **Grey Heron**  ***Ardea cinerea*** | **Eurasian Bittern *Botaurus stellaris*** | **Little Egret**  ***Egretta garzetta*** | **Medium Egret**  ***Ardea intermedia*** | **Night Heron**  ***Nycticorax nycticorax*** | **Purple Heron**  ***Ardea purpurea*** | **Reddish Egret**  ***Egretta rufescens*** |
| --- | --- | --- | --- | --- | --- | --- | --- | --- | --- | --- | --- |
| mean total body mass [kg] | 0.505 ^[1]^ | 0.346 ^[2]^ | 0.475 ^[3]^ | 0.888 ^[4]^ | 1.439 ^[4]^ | 1.133 ^[4]^ | 0.505 ^[4]^ | 0.400 ^[3]^ | 0.763 ^[4]^ | 0.906 ^[4]^ | 0.450 ^[3]^ |
| wingspan [m] | 1.00 ^[5]^ | 0.93 ^[2]^ | 1.06 ^[3]^ | 1.55 ^[3]^ | 1.65 ^[3]^ | 1.15 ^[3]^ | 0.92 ^[2]^ | 1.1 ^[3]^ | 0.95 ^[3]^ | 1.35 ^[3]^ | 1.17 ^[3]^ |
| mean wingspan [m] | n.d. | n.d. | n.d. | 1.44 ^[4]^ | 1.73 ^[4]^ | 1.26 ^[4]^ | n.d. | n.d. | 1.06 ^[4]^ | n.d. | n.d. |
| wing area [m**^2^**] | n.d. | n.d. | n.d. | 0.2443 ^[3]^ | 0.3716 ^[3]^ | 0.2727 ^[3]^ | n.d. | n.d. | 0.1589 ^[3]^ | 0.2488 ^[3]^ | n.d. |
| wing load [kg/m**^2^**] | n.d. | n.d. | n.d. | 3.63 ^[4]^ | 3.87 ^[4]^ | 4.15 ^[4]^ | n.d. | n.d. | 4.80 ^[4]^ | 3.64 ^[4]^ | n.d. |
| Region | French Guiana ^[6]^ | S Africa ^[7]^ | E China ^[8,9]^; Korea ^[10]^ | Australia ^[11]^; W USA ^[12]^; C Poland ^[13]^ | SE China ^[14]^; Korea ^[15,16]^; Far East Russia ^[17]^; Poland ^[18]^ | E China ^[19]^; The Netherlands ^[20]^ | Australia ^[11]^; SE China ^[21]^ | Australia ^[11]^ | Poland ^[22]^ | The Netherlands ^[23]^ | S USA ^[24,25]^ |
| Age (number of ind.) | ad. (4) ^[6]^ | ad. and juv. 481) ^[7]^ | ad. (60) ^[8]^;  juv. (2) ^[10]^;  juv. (33) ^[9]^ | ad. (11) ^[12]^;  ad. and juv. (51) ^[13]^; juv. (5) ^[11]^ | ad. (1) ^[14]^;  ad. (4) ^[15]^; juv. (8) ^[17]^; juv. (1) ^[16]^; ad. and juv. ^[18]^ | n.d. about age (2) ^[19]^;  ad. (10) ^[20]^ | ad. (1) and juv. (1) ^[21]^;  juv. (6) ^[11]^ | juv. (10) ^[11]^ | ad. (3) ^[22]^ | ad. (5) and juv. (3) ^[23]^ | juv. (24) ^[24]^; ad. (30) ^[25]^ |
| Approach | GPS/  GSM ^[6]^ | ring recoveries ^[7]^ | GPS/  GSM ^[8-10]^ | ring recoveries ^[11,13]^; GPS/  GSM ^[12]^ | GPS/  GSM ^[14-17]^;  ring recoveries ^[18]^ | GPS/  GSM ^[19]^ | GPS/  GSM ^[21]^; ring recoveries ^[11]^ | ring recoveries ^[11]^ | GPS/  GSM ^[22]^ | GPS/  GSM ^[23]^ | satellite-received transmitter ^[24]^; GPS/  GSM ^[25]^ |
| AM Duration [days] | 27.3±13.3 ^[6]^ | 115 ^[7]^ | 16.2±16.9 ^[8]^;  4.5±0.7 ^[10]^;  14.7 ^[9]^ | n.d. | 5.71 ^[14]^;  8.0±5.7 ^[15]^; 10.1±11.2 ^[17]^; 41 ^[16]^ | 26.8±35.9 ^[20]^; 47.8±8.9 ^[19]^ | 5.5±4.2 ^[21]^ | n.d. | 35.0±29.5 ^[22]^ | 6.91±2.7 ^[23]^ | n.d. |
| SM Duration [days] | n.d. | n.d. | 43.5±20.2 ^[8]^ | n.d. | 4.1±2.1 ^[14]^; 19.4±15.1 ^[17]^ | 5.0±5.1 ^[20]^; 58.3±13.2 ^[19]^ | 3.2±0.4 ^[21]^ | n.d. | n.d. | n.d. | n.d. |
| AM Distance [km] | 647.0±526.3 ^[6]^ | 1346.3±248.7 ^[7]^ | 4,047.6±775.7 ^[8]^;  2,564.5±1,069.9 ^[10]^;  3,175.0±1,171.0 ^[9]^ | 883±200 ^[13]^;  273±491 ^[11]^;  293±109 ^[12]^ | 3,003.0 ^[14]^  1,391.7±392.5 ^[15]^; 2,951.9±1,685.0 ^[17]^;  1,205±224.9 ^[18]^ | 1,500.0±1,510.5 ^[20]^; 3,828.7±433.6 ^[19]^ | 1043.5±0.7 ^[21]^;  1502±1298 ^[11]^ | 1020±990.9 ^[11]^ | 1,667.0±144.3 ^[22]^ | 4,088.3±706.9 ^[23]^ | 241.3±305.3 ^[24]^ |
| SM Distance [km] | n.d. | n.d. | 4,767.6±668.5 ^[8]^ | n.d. | 2,781.5±99.7 ^[14]^; 3,187.0±1,498.0^[17]^ | 1,948.0±2171.4 ^[20]^; 4,127.1±527.0 ^[19]^ | 1046.7±83.0 ^[21]^ | n.d. | n.d. | n.d. | n.d. |
| AM speed [km/h] | n.d. | n.d. | 60.0±12.2 ^[8]^ | n.d. | n.d. | n.d. | 26.3±19.1 ^[21]^ | n.d. | 54.5 ^[22]^ | 643.9±194.0 ^[23]^ | n.d. |
| SM speed [km/h] | n.d. | n.d. | 46.2±7.9 ^[8]^ | n.d. | 32.2±15.3 ^[14]^ | n.d. | 36.2±7.9 ^[21]^ | n.d. | n.d. | n.d. | n.d. |
| AM speed [km/day] | 23.3±11.5 ^[6]^ | n.d. | 249.9 ^[8]^ | n.d. | 525.9 ^[14]^; 174.0 ^[15]^; 292.3 ^[17]^ | 56.0 ^[20]^ | 189.7 ^[21]^ | n.d. | 47.6 ^[22]^ | 591.7 ^[23]^ | n.d. |
| SM speed [km/day] | n.d. | n.d. | 109.7 ^[8]^ | n.d. | 678.4 ^[14]^; 164.3 ^[17]^ | n.d. | 332.3 ^[21]^ | n.d. | n.d. | n.d. | n.d. |
| AM No. of Stop-over Sites | 1.0±0.0 ^[6]^ | n.d. | 3.5 ±1.4 ^[8]^;  3.0±1.4 ^[10]^ | n.d. | 0 ^[14]^, 8.0±2.8 ^[15]^;  8.0±1.6 ^[17]^; 1 ^[16]^ | 4±3.1 ^[20]^; 2.8±1.0 ^[19]^ | 2.0±0.0 ^[21]^ | n.d. | 2.5±2.1 ^[22]^ | n.d. | 10.3±10.9 ^[25]^ |
| SM No. of Stop-over Sites | n.d. | n.d. | 8.7±2.6 ^[8]^ | n.d. | n.d. | 2.7±2.1 ^[20]^; 3.5±1.3 ^[19]^ | 1±0.0 ^[21]^ | n.d. | 4.0 ^[22]^ | n.d. | 11.5±6.4 ^[25]^ |
| AM Stop-over Duration [days] | 36.3±21.4 ^[6]^ | n.d. | 1.0±0.0 ^[10]^ | n.d. | 0 ^[14]^; 2.3±7.4 ^[17]^; 39 ^[16]^ | 2.2 ^[20]^; 55.5±15.1 ^[19]^ | n.d. | n.d. | 13.4±4.9 ^[22]^ | n.d. | 5.7±6.5 ^[25]^ |
| SM Stop-over Duration [days] | n.d. | n.d. | 50.6±22.9 ♀; 36.4±15.4 ♂ ^[8]^ | n.d. | 0 ^[9]^ | 12.8 ^[20]^; 71.3±16.2 ^[19]^ | n.d. | n.d. | 17.0 ^[22]^ | n.d. | 9.1±20.6 ^[25]^ |

References:

[1] Hancock J, Kushlan JA. *The Herons Handbook*, A&C Black: London, UK; 1984.

[2] Voisin C. *The herons of Europe*. T & A D POYSER, London, UK; 1991.

[3] Del Hoyo J, Elliot A, Sargatal J, Christie DA. *Handbook of the birds of the world*, Lynx Ed., Barcelona, Vol. 1, No. 8; 1992.

[4] Alerstam T, Rosén M, Bäckman J, Ericson PGP, Hellgren O. Flight speeds among bird species: allometric and phylogenetic effects. PLoS Biol 2007;5, e197. doi:10.1371/journal.pbio.0050197

[5] animaldiversity.org (accessed on May 2025)

[6] Stier A, Ricardou A, Uriot S, De Pracontal N, Kushlan JA. Breeding season home range and migration of the Agami Heron (*Agamia agami*). Waterbirds 2017;289–296.

[7] Kopij G. Migratory connectivity of south african cattle egrets (*Bubulcus ibis*, Ciconiiformes, Ardeidae). Zool Zhurnal 2017;96:418–428.

[8] Huang Z, Zhou X, Fang W, Chen X. Migration and wintering of vulnerable adult Chinese Egrets (*Egretta eulophotes*) revealed by GPS tracking. Avian Res 2022;13;100055. https://doi.org/10.1016/j.avrs.2022.100055

[9] Xu H, Yang Z, Liu D, Jia R, Chen L, Liang B, Zhang Z, Zhang, G. Autumn migration routes of fledgling Chinese Egrets (*Egretta eulophotes*) in Northeast China and their implications for conservation. Avian Res 2022;13;100018. https://doi.org/10.1016/j.avrs.2022.100018

[10] Son SJ, Lee SK, Oh JW, Kim IK, Kim DH, Hyun BR, Kang J. H. First record of autumn migration of juvenile Chinese egrets *Egretta eulophotes* from South Korea tracked with GPS telemetry. Pak J Zool 2022;54.

[11] Geering DJ, Maddock M, Cam GR, Ireland C, Halse SA, Pearson GB. Movement patterns of Great, Intermediate and Little Egrets from Australian breeding colonies. Corella 1998;22**:**37–45.

[12] Lumpkin D, Jennings S, Warnock N, Condeso TE. Partial Migration by Great Egrets *Ardea alba* in Coastal California. Waterbirds 2003;4:150–158. https://doi.org/10.1675/063.045.0205

[13**]** Włodarczyk R, Szafara D, Kaczmarek K, Janiszewski T, Minias P. Migratory behaviour and survival of Great Egrets after range expansion in Central Europe. PeerJ 2020;8;e9002.

[14] Ye X, Xum Z, Aharon-Rotman Y, Yu H, Cao L. First description of Grey Heron *Ardea cinerea* migration recorded by GPS/GSM transmitter. Ornithol *Sci* 2018;17:223–228. https://doi.org/10.2326/osj.17.223

[15] Lim EH, Shin MS, Cho HJ, Kim IK, Shin YU, Oh HS, Lee EJ. Migration and Home Range of the Grey Heron (*Ardea cinerea*) in the Republic of Korea. Waterbirds 2021;44:93–97. https://doi.org/10.1675/063.044.0109

[16] Kim DW, Kim HJ, Kwon IK, Hwang JW, Kim JH. Post-breeding dispersal and autumn migration of a juvenile Grey Heron (*Ardea cinerea*). Kor J Orni 2015;22:67–72.

[17] Tiunov I, Katin I, Lee KN, Hong SK, Lee I, Yoon H, Lee E, Lee S. Migration of Grey Heron from the Peter the Great Bay and the potential transmission routes of avian influenza. J Asia-Pacific Biodiv 2022;15:157–164.

[18] Manikowska-Ślepowrońska B, Mokwa T, Jakubas D. Wintering and Stop-Over Areas of Grey Herons (*Ardea cinerea*) Breeding in Central Europe: A Ring-Recovery Analysis. Ann Zool Fenn 2018;55:277–285. https://doi.org/10.5735/086.055.0601

[19] Gu D, Chai Y, Gu Y, Hou J, Cao L, Fox AD. Annual migration routes, stopover patterns and diurnal activity of Eurasian Bitterns *Botaurus stellaris* wintering in China. Bird Study 2019;66:43–52. doi: 10.1080/00063657.2019.1608906

[20] van der Winden J, Hogeweg N, Baaij E, van Horssen PW, Shamoun-Baranes J, Vos R, Piersma T. The influence of weather on the migration behaviour of Eurasian Bitterns *B*o*taurus stellaris*. Bird Study 2021;68:370–380. https://doi.org/10.1080/00063657.2022.2095350.

[21] Pang CC, Sung YH, Chung YT, Ying HK, Fong HNH, Yu YT. Full migration routes of two Little Egrets (*Egretta garzetta*) display breeding and wintering site fidelity. Ornithol Sci 2023;22:81–86.

[22] Ledwoń M, Betleja J. Post-breeding migration of Night Herons *Nycticorax nycticorax* tracked by GPS/GSM transmitters. J Ornithol 2014;156:313–316. DOI 10.1007/s10336-014-1131-z

[23] van der Winden J, Poot MJM, Van Horssen P. W. Large birds can migrate fast: The post-breeding flight of the Purple Heron *Ardea purpurea* to the Sahel. Ardea 2010;98:395–402. https://doi.org/10.5253/078.098.0313.

[24] Geary B, Green MC, Ballard BM. Movements and survival of juvenile reddish egrets *Egretta rufescens* on the Gulf of Mexico coast. Endanger Species Res 2015;28:123–134.

[25] Koczur L, Ballard B. Migratory stopover sites used by Reddish Egrets: prioritization for conservation. Avian Conserv Ecol 2022;17:15. doi:10.5751/ACE-01973-170115
